# *Quadriacanthus* species (Monogenea: Dactylogyridae) from catfishes (Teleostei: Siluriformes) in eastern Africa: new species, new records and first insights into interspecific genetic relationships

**DOI:** 10.1186/s13071-017-2223-4

**Published:** 2017-08-01

**Authors:** Kateřina Francová, Mária Seifertová, Radim Blažek, Milan Gelnar, Zuheir N. Mahmoud, Eva Řehulková

**Affiliations:** 1 Department of Botany and Zoology, Faculty of Science, Masaryk University, Kotlářská 2, 611 37 Brno, Czech Republic; 2 Institute of Vertebrate Biology, Academy of Sciences of the Czech Republic, Květná 8, 603 65 Brno, Czech Republic; 3 Department of Zoology, Faculty of Science, University of Khartoum, Khartoum, Sudan

**Keywords:** Monogenea, Dactylogyridae, *Quadriacanthus*, Siluriformes, Catfishes, Africa, Lake Turkana, Nile River Basin, New species, DNA

## Abstract

**Background:**

African catfishes of the families Bagridae and Clariidae are known to be parasitized with monogeneans of *Quadriacanthus* Paperna, 1961 (Dactylogyridae). The genus remains taxonomically challenging due to its speciose nature and relatively wide host range representing two fish orders, i.e. Siluriformes and Osteoglossiformes, in Africa and Asia. Here, we investigated diversity of *Quadriacanthus* spp. parasitizing *Clarias gariepinus* (Burchell), *Heterobranchus bidorsalis* Geoffroy Saint-Hilaire, and *Bagrus docmak* (Forsskål) collected in the Lake Turkana (Kenya) and Nile River Basin (Sudan). The interspecific relationships among *Quadriacanthus* spp. parasitizing catfishes inferred from ribosomal DNA sequences were investigated for the first time.

**Methods:**

A combined morphological and molecular approach was used for description of the new species and for a critical review of the previously described *Quadriacanthus* spp., by means of phase contrast microscopic examination of sclerotized structures, and assessing the genetic divergence among the species found using rDNA sequences.

**Results:**

Seven species (including four new) of *Quadriacanthus* were identified. These were as follows: *Quadriacanthus aegypticus* El-Naggar & Serag, 1986, *Quadriacanthus clariadis* Paperna, 1961, *Quadriacanthus fornicatus* n. sp., *Quadriacanthus pravus* n. sp., and *Quadriacanthus zuheiri* n. sp. from *Clarias gariepinus* (Clariidae); *Quadriacanthus mandibulatus* n. sp. from *Heterobranchus bidorsalis* (Clariidae); and *Quadriacanthus bagrae* Paperna, 1979 from *Bagrus docmak* (Bagridae). For both 18S-ITS1 and 28S rDNA regions, *Q. clariadis* from a clariid fish was found to be most closely related to *Q. bagrae* from a bagrid host*. Quadriacanthus mandibulatus* n. sp. was observed to be the most distant species from the others. The separation of *Q. mandibulatus* n. sp. from the other species corresponds with the different morphology of its copulatory tube. The copulatory tube is terminally enlarged in *Q. mandibulatus* n. sp*.*, while the tube in all other congeners studied is comparatively small and with an oblique tapering termination.

**Conclusions:**

This study contributes to a better understanding of African dactylogyrid diversity and provides the first molecular characterization of *Quadriacanthus* spp. The observed interspecific genetic relationships among *Quadriacanthus* spp. from clariids and *Q. bagrae* from a bagrid host suggest a possible host-switching event in the evolutionary history of the genus. Our records extend the currently known geographical range for *Quadriacanthus* spp. to Kenya and Sudan.

## Background

Monogenea is a diverse group of mostly ectoparasitic flatworms showing great potential as model organisms to study the ecological and evolutionary processes that drive diversification and speciation. The high host specificity shown by most monogeneans enables searches for links between the ecological characteristics of the hosts and the diversity of their parasites [1].

Among monogeneans, *Quadriacanthus* Paperna, 1961 (Dactylogyridae) represents one of the genera with wider host and geographical distribution. Although this genus comprises mostly gill parasites of African and Asian clariids (Siluriformes, Clariidae), one species has been recorded on bagrids (Siluriformes, Bagridae) and one species on phylogenetically distant notopterids (Osteoglossiformes, Notopteridae) in Africa [2, 3].

The genus was proposed by Paperna [4] for *Q. clariadis* Paperna, 1961 from the gills of *Clarias gariepinus* (Burchell) (syn. *C. lazera*) collected in Israel and characterized, in part, by having two unequal bars, each with a solid base. Kritsky & Kulo [5] subsequently emended the diagnosis of *Quadriacanthus* and recognized that the ventral bar is composed of two components articulating medially. Despite the work of these authors, Dubey et al. [6] established *Anacornuatus* Dubey, Gupta & Agarwal, 1992 for those species of *Quadriacanthus* that possess a two-piece ventral bar instead of a single-piece ventral bar, as indicated by Paperna [4]. They were evidently unaware of the work of Kritsky & Kulo [5] and hence erred in proposing the new genus. Consequently, Lim et al. [2], who listed 24 species of *Quadriacanthus* parasitizing Clariidae (*Clarias* spp. and *Heterobranchus* spp.) and African *Bagrus* spp., plus one *Quadriacanthus* species of doubtful validity, infecting tilapia (see also [5]), synonymized *Anacornuatus* with *Quadriacanthus*. However, these authors were not able to ascertain the validity of the two species assigned to *Anacornuatus* and considered them as *species inquirendae*. Tripathi et al. [7] added generic characters, redefined the dorsal bar as “T or Y-shaped with mid-posterior process”, and limited the taxon to 25 species (including *Anacornuatus postbifidus* Dubey, Gupta & Agarwal, 1992 as a new combination within *Quadriacanthus*). The recent descriptions of three new species from *Clarias submarginatus* Peters by Bahanak et al. [8] brings the number of *Quadriacanthus* species from siluriform hosts to 28. Nack et al. [3] revealed the presence of a species of *Quadriacanthus* on a fish host belonging to the Notopteridae (Osteoglossiformes). This finding extends the host range of species of *Quadriacanthus* to a new family (Notopteridae) and even a new order (Osteoglossiformes).

In a recent survey of monogeneans parasitizing catfishes from Kenya and Sudan, we recovered three described (*Q. aegypticus* El-Naggar & Serag, 1986, *Q. clariadis* Paperna, 1961 and *Q. bagrae* Paperna, 1979) and four new species of *Quadriacanthus*. We thus aimed to describe these four species and determine their relationships to congeners based on the partial 18S, entire ITS1, and partial 28S rDNA sequences.

## Methods

### Fish collection

**Fig. 1:**
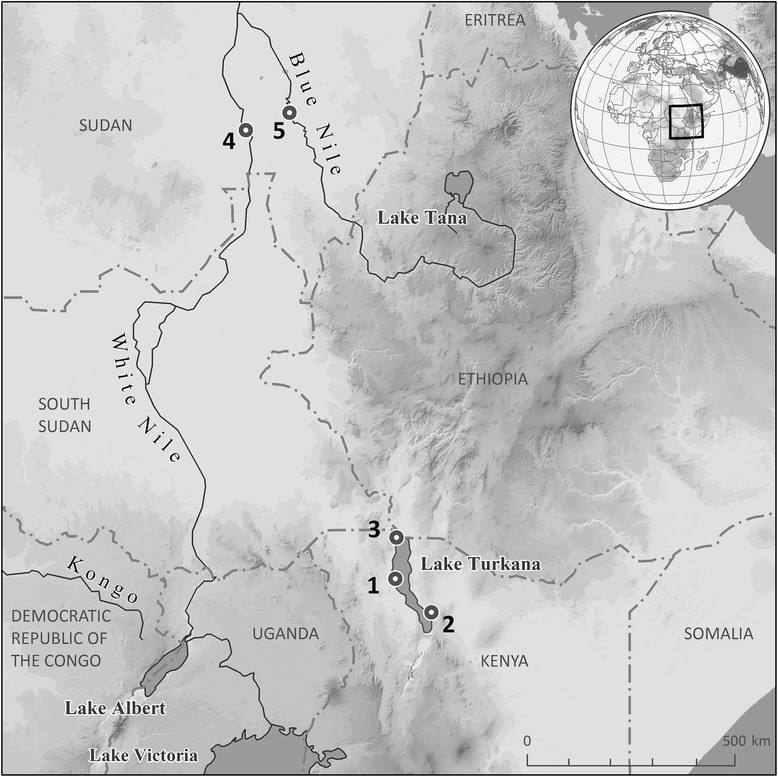
Map of the localities from which siluriform species were collected during 2008–2014 (see also Table 1)

Catfish hosts *Bagrus docmak* (Forsskål) (Bagridae), *Clarias gariepinus* (Burchell) and *Heterobranchus bidorsalis* Geoffroy Saint-Hilaire (Clariidae) (all autochthonous fishes) were collected by hook-and-line or beach seine net, or purchased at local fish markets in five localities in Kenya (Lake Turkana) and Sudan (White and Blue Nile River) during the period 2008–2014 (Table 1; Fig. 1). Fish hosts were identified using the keys given by Bailey [9] and Hopson & Hopson [10]. Scientific and common names of fishes are those provided in FishBase [11] and verified in Eschmeyer et al. [12].

**Table 1:** Localities from which siluriform species were collected during 2008-2014

Locality number	Locality name	Coordinates	Year of collection
1	Kalokol - Fishing Lodge, Lake Turkana, Kenya	3°33′18.26″N, 35°54′56.03″E	2008, 2009
2	Loyingalani - El Molo Bay, Lake Turkana, Kenya	2°49′45.55″N, 36°41′55.32″E	2008, 2009
3	Todonyang - Omo River Delta, Lake Turkana, Kenya	4°27′6.37″N, 35°56′15.44″E	2008, 2009
4	Fishmarket in Kosti, White Nile, Sudan	13°10′18.58″N, 32°40′19.24″E	2010, 2014
5	Fishmarket in Sennar, Blue Nile, Sudan	13°32′31.09″N, 33°37′15.79″E	2010, 2014

### Parasite collection and identification

The gills of freshly killed fishes were extracted and examined in bottled water under a dissecting microscope. Live monogeneans were individually picked from the gills with fine needles and immediately processed. Some specimens were prepared for morphological studies following Musilová et al. [13]. Briefly, they were flattened using coverslip pressure in order to best expose their hard parts, and fixed with a mixture of glycerine and ammonium picrate (GAP). Specimens collected for DNA analyses were bisected using fine needles under a dissecting microscope. Subsequently, one half of the body (either the posterior part with haptoral sclerites or anterior part containing the male copulatory organ) was fixed in 96% ethanol for later molecular analysis; the other body half was completely flattened under coverslip pressure and fixed with GAP for species identification. The body half in GAP was deposited (one per species) as a hologenophore, i.e. a voucher specimen from which a molecular sample is directly derived (see [14] for terminology). Parasite specimens collected in Kenya were not used for molecular analysis.

**Fig. 2:**
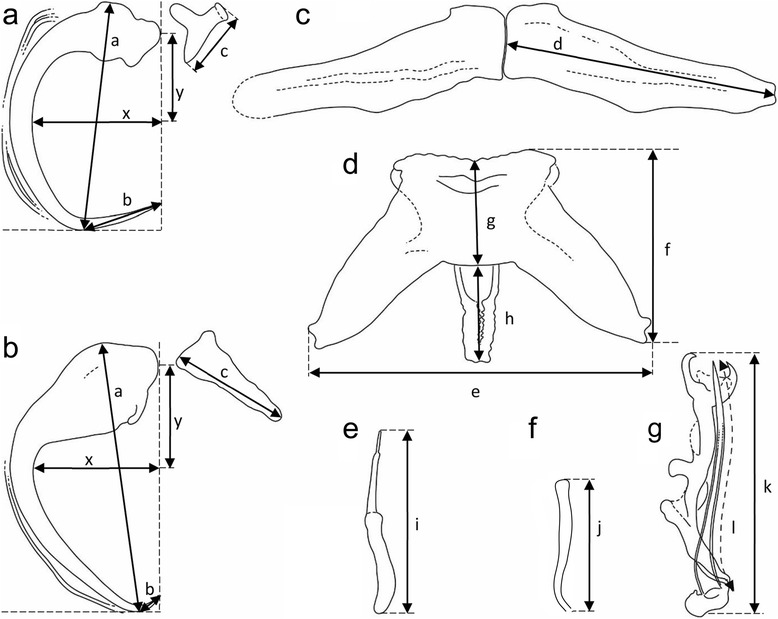
Scheme of measurement for sclerotized structures of *Quadriacanthus* spp. **a** Ventral anchor. **b** Dorsal anchor (*a*, total length; *b*, point length; *c*, total length of accessory sclerite; x/y, curvature of shaft). **c** Ventral bar (*d*, component length). **d** Dorsal bar (*e*, total length; *f*, total width excluding the process; *g*, thickness at mid-region; *h*, process length). **e** Hook (*i*, total length). **f** Vagina (*j*, total length). **g** MCO (*k*, total length; *l*, trace length of copulatory tube)

The mounted monogenean specimens (or their parts) were studied using an Olympus BX 61 microscope equipped with phase contrast optics, and drawings were made with the aid of a drawing attachment. Measurements, all in micrometres, were taken using digital image analysis (Stream Motion, version 1.9.2) and are presented as the range followed by the mean and the number (*n*) of specimens measured in parentheses. The dimensions of the body and haptor were obtained from unflattened specimens as the longest measurements in dorsoventral view; measurements of the sclerotized structures (the haptoral and reproductive hard parts) were taken from specimens flattened under coverslip pressure, facilitated by the blotting of excess water with a filter paper. The schemes of measurement for the hard structures are shown in Fig. 2; in essence, the method of measuring the anchors follows the procedures outlined by Řehulková & Gelnar [15]. The numbering of hook pairs (in Roman numerals) follows that recommended by Mizelle [16]. The male copulatory organ is henceforth abbreviated to MCO. For comparative purposes, specimens of some previously described species were examined: *Quadriacanthus agnebiensis* N’Douba, Lambert & Euzet, 1999 (MNHN 572 HF Tk 89); *Quadriacanthus clariadis* Paperna, 1961 (MRAC 37.160); *Quadriacanthus numidus* Kritsky & Kulo, 1988 (MNHN 146 HF); *Quadriacanthus thysi* N’Douba, Lambert & Euzet, 1999 (MNHN 577 HF Tk 94 and 576 HF Tk 93; and MRAC 37416). Note that the authorities of the new taxa described below are Francová & Řehulková (according to International Code of Zoological Nomenclature [17]).

### DNA extraction, PCR amplification and sequencing

DNA was extracted from 2 to 6 individuals of each collected species using a DNeasy® Blood & Tissue Kit (Qiagen, Hilden, Germany) following the manufacturer’s instructions. DNA was stored in AE buffer at -20 °C. Two nuclear ribosomal DNA fragments were used in our analysis: fragment spanning partial 18S rDNA (18S) and entire internal transcribed spacer 1 (ITS1), and fragment of partial nuclear 28S rDNA (28S). Until now, only two 28S sequences for *Quadriacanthus kobiensis* Ha, 1968 (EF100545, AY841874) and one 18S-ITS1 fragment for *Quadriacanthus* sp. (HG491496) had been deposited in GenBank. The partial 28S fragment was amplified using primers C1 (forward; 5′-ACC CGC TGA ATT TAA GCA T-3′) and D2 (reverse; 5′-TGG TCC GTG TTT CAA GAC-3′) [18]. The 18S-ITS1 fragment was amplified in one round using primers S1 (forward, 5′-ATT CCG ATA ACG AAC GAG ACT-3′) [19] and IR8 (reverse, 5′-GCT AGC TGC GTT CTT CAT CGA-3′), that anneal to the 18S and 5.8S rDNA genes, respectively [20]. PCRs were performed according to Mendlová et al. [21]. The PCR products were electrophoresed on a Gold View strained agarose gel (2%) and then successful PCRs, in which a single fragment was amplified, were purified using High Pure PCR Product Purification Kit (Roche, Mannheim, Germany). The purified PCR products were sequenced for both strands with the same primers as used in the amplification. Sequencing was carried out using BigDye® Terminator v3.1 Cycle Sequencing Kit and an Applied Biosystems 3130 Genetic Analyzer. Nucleotide sequences of the 18S-ITS1 and partial 28S regions were assembled and edited using Sequencher software (Gene Codes Corporation, Ann Arbor, MI, USA). The final sequences were deposited in the GenBank database under accession numbers KX713993–KX713998 and KX685951–KX685956.

### Sequence and phylogenetic analysis

Because no significant differences were found between 18S and ITS1 sequence data (partition homogeneity test, *P* = 1.00), further analyses were performed based on concatenated 18S-ITS1 sequences. Two datasets (18S-ITS1 and 28S) were used to estimate the interspecific relationships among the *Quadriacanthus* species. All corrected 18S-ITS1 and 28S sequences were aligned using ClustalW [22] and improved manually using the program BioEdit version 7.1.11 [23]. Alignments were then trimmed automatically using TrimAl v.1.3 [24]. Calculations of genetic distances (Kimura 2-parameter [25]) among sequences of *Quadriacanthus* species were carried out in MEGA 7 [26].

Maximum likelihood analyses were conducted using MEGA 7 [26] with 1000 rapid bootstrap replicates. Data were modelled according to the K2 + G model. Phylogenetic trees were rooted by including available data from GenBank: *Schilbetrema* sp. (HG491495) isolated from *Schilbe intermedius* Rüppell in Africa for the 18S-ITS1 dataset, and *Schilbetrema* sp. (KP056243) isolated from *Pareutropius debauwi* (Boulenger) in West Africa for the 28S dataset. *Quadriacanthus* sp. (HG491496) isolated from *Heterobranchus bidorsalis* Geoffroy Saint-Hilaire in Senegal (West Africa) was also included in the 18S-ITS1 phylogenetic analysis, and *Q. kobiensis* Ha, 1968 (AY841874) isolated from *Clarias batrachus* (Linnaeus) in China was used in the 28S phylogenetic analysis.

## Results

Our investigation of the monogeneans from the gills of three catfish species revealed the presence of three previously described and four new species of *Quadriacanthus*. *Bagrus docmak* (Bagridae) and *Heterobranchus bidorsalis* (Clariidae) were each found to harbour one *Quadriacanthus* species, *Q. bagrae* and *Q. mandibulatus* n. sp., respectively. Five *Quadriacanthus* species were found to infect *Clarias gariepinus* (Clariidae): *Q. aegypticus*, *Q. clariadis*, *Q. fornicatus* n. sp., *Q. pravus* n. sp., and *Q. zuheiri* n. sp. All the species found are morphologically characterized and described below.

**Family Dactylogyridae Bychowsky, 1933**

**Genus** ***Quadriacanthus*** **Paperna, 1961**

***Quadriacanthus aegypticus*** **El-Naggar & Serag, 1986**

Syn. *Anacornuatus aegypticus* (El-Naggar & Serag, 1986) Dubey, Gupta & Agarwal, 1992

***Type-host and locality*** **:** *Clarias gariepinus* (Burchell) (syn. *C. lazera*) (Clariidae), Lake Manzala and the Demietta branch of the River Nile, Dakahlia Governorate, Egypt [27].

***Host*** **:** *Clarias gariepinus* (present study).

***Localities*** **:** Lake Turkana, Kenya (localities 1–3); Nile River Basin, Sudan (localities 4, 5) (present study).

***Site in host*** **:** Gill lamellae.

***Other records*** **:** *C. gariepinus* (syn. *C. lazera*), Nile River near Cairo, Egypt [5]; *C. gariepinus*, Lake Kariba, Zimbabwe [28]; *C. gariepinus*, River Nile in the Helwan locality, south Cairo, Egypt [29]; *C. gariepinus*, Nwanedi-Luphephe Dams, Limpopo River System, South Africa [30]; *C. gariepinus*, Vaal Dam, Gauteng Province, South Africa [31]; *C. gariepinus*, Lake Tana (Bahir Dar), Ethiopia [32].

***Voucher material*** **:** MNHN HEL625 (1 specimen; locality 4); MNHN HEL626 (2 specimens; locality 1); MNHN HEL627 (1 specimen; locality 3); IPCAS M-632 (1 specimen from locality 1; 1 specimen from locality 5).

### Measurements

**Fig. 3:**
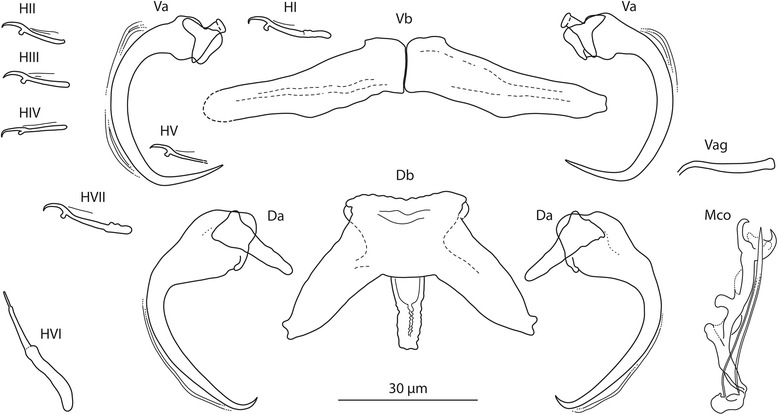
*Quadriacanthus aegypticus* El-Naggar & Serag, 1986 ex *C. gariepinus* (Kenya). Sclerotized structures. *Abbreviations*: Va, ventral anchor; Vb, ventral bar; Da, dorsal anchor; Db, dorsal bar; HI-HVII, hooks; Vag, vagina; Mco, male copulatory organ

[Based on 10 flattened and 3 unflattened specimens in GAP; Fig. 3]. Body length 475–569 (513; *n* = 3); greatest width 80–125 (108; *n* = 3). Haptor 95–100 (97; *n* = 3) long, 110–155 (136; *n* = 3) wide. Ventral anchor: a = 33–37 (35; *n* = 10); b = 11–13 (12; *n* = 10); c = 9–11 (10; *n* = 10); x/y = 1.2–1.7 (1.4; *n* = 10). Dorsal anchor: a = 40–44 (42; *n* = 10); b = 3–5 (4; *n* = 10); c = 16–21 (19; *n* = 10); x/y = 1.1–1.2 (1.1; *n* = 10). Ventral bar: d = 40–46 (43; *n* = 10). Dorsal bar: e = 39–52 (49; *n* = 10); f = 21–31 (28; *n* = 10); g = 10–17 (15; *n* = 10); h = 14–17 (15; *n* = 10). Hooks: 7 pairs; *i* = 11–29 (17; *n* = 10): hook I 16–19 (17; *n* = 10); hooks II–V 11–15 (13; *n* = 10); hook VI 26–29 (29; *n* = 10); hook VII 18–19 (18; *n* = 10). Vagina: slightly sclerotized; j = 17–21 (19; *n* = 10). MCO: k = 39–44 (41; *n* = 10); l = 36–40 (38; *n* = 10).

### Differential diagnosis

The present specimens closely fit the characters of the original description of *Q. aegypticus* by El-Naggar & Serag [27] as well as the drawings/measurements provided by Kritsky & Kulo [5] and Douëllou & Chishawa [28] and there is little doubt that they are conspecific. However, it should be mentioned that in the original paper by Douëllou & Chishawa [28] the illustrations of *Q. aegypticus* were mixed with those of another *Quadriacanthus* species; their figure 2 (identified as the haptoral structures, MCO and vagina of *Q. aegypticus*) clearly represents the sclerotized structures (actually without depiction of a vagina) of another species of *Quadriacanthus*, most probably those of *Q. clariadis*, although the morphometrical characteristics of the sclerotized structures correspond to those originally described for *Q. aegypticus*. Also, Douëllou & Chishawa's [28] figure 1 (identified as the sclerotized structures of *Q. bagrae*) and figure 2 are identical (see also remarks to *Q. bagrae*); indeed, figure 2 was later replaced with the correct version (i.e. illustrating the sclerotized structures of the real *Q. aegypticus*) as an erratum to Douëllou & Chishawa's [28] original paper.

*Quadriacanthus aegypticus* is most similar to *Q. clariadis* on the basis of the morphology of the haptoral sclerites. It differs from the latter species by having (i) noticeably larger ventral anchors in relation to the ventral bar; (ii) a ventral anchor with an elongated shaft (the shaft of the ventral anchor is comparatively shorter in *Q. clariadis*); (iii) a sclerotized vagina; and (iv) an accessory piece with two claw-like hooks serving as a guide for the distal portion of the copulatory tube and a medial part modified into two protruding diverticula.

***Quadriacanthus bagrae*** **Paperna, 1979**

Syn. *Quadriacanthus clariadis bagrae* Paperna, 1979

***Type-host and locality*** **:** *Bagrus docmak* (Forsskål) (syn. *B. docmac*) (Bagridae), Lake Victoria, Uganda [33].

***Host*** **:** *Bagrus docmak* (present study).

***Locality*** **:** Nile River Basin, Sudan (locality 5) (present study).

***Site in host*** **:** Gill lamellae.

***Other records*** **:** *Bagrus docmak* (syn. *B. docmac*), Albert-Nile near Chobe, Uganda [33]; *Bagrus bajad* (syn. *B. bayad*)*,* Lake Albert, Uganda [33]; *Bagrus orientalis*, River Ruaha, Tanzania [33]; *Clarias gariepinus* (syn. *C. lazera*), River Nile near Cairo, Egypt [5]; *Clarias gariepinus*, Lake Kariba, Zimbabwe [28]; *C. gariepinus*, River Gomti, Lucknow, State of Uttar Pradesh, India [7].

***Voucher material*** **:** MNHN HEL628 (1 specimen; locality 5); MNHN HEL629 (1 specimen; locality 5); IPCAS M-633 (2 specimens; locality 5). Hologenophore: MNHN HEL641 (locality 5).

***Representative DNA sequences*** **:** 18S-ITS1 rDNA (GenBank acc. no. KX713993) and 28S rDNA (GenBank acc. no. KX685951) (see also Table 2).

**Table 2:** List of *Quadriacanthus* species used in this study, including their host species, locality (with number in parentheses), total number of isolates and GenBank accession numbers for 18S-ITS1 and 28S sequences

Parasite species	Host species	Locality of collection^a^	Isolates	18S-ITS1	28S
*Q. aegypticus*	*Clarias gariepinus*	LT (1, 2, 3); WN (4); BN (5)	–	–	–
*Q. bagrae*	*Bagrus docmak*	**BN (5)**	3	KX713993	KX685951
*Q. clariadis*	*Clarias gariepinus*	LT (1, 3); **WN (4)**; **BN (5)**	6	KX713994	KX685952
*Q. fornicatus* n. sp.	*Clarias gariepinus*	**WN (4)**; **BN (5)**	3	KX713995	KX685953
*Q. mandibulatus* n. sp.	*Heterobranchus bidorsalis*	LT (3); **BN (5)**	5	KX713996	KX685954
*Q. pravus* n. sp.	*Clarias gariepinus*	WN (4); **BN (5)**	3	KX713997	KX685955
*Q. zuheiri* n. sp.	*Clarias gariepinus*	WN (4); **BN (5)**	2	KX713998	KX685956

*Abbreviations*: *LT* Lake Turkana, Kenya, *WN* White Nile (Kosti), Sudan, *BN* Blue Nile (Sennar), Sudan

^a^Localities where specimens were collected for molecular analysis are shown in bold

### Measurements

**Fig. 4:**
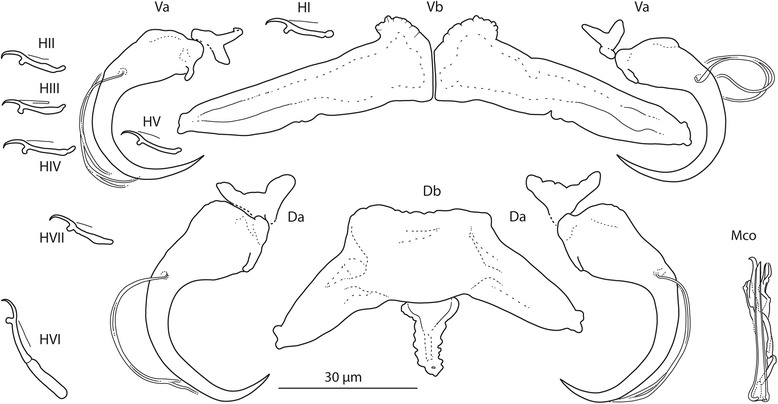
*Quadriacanthus bagrae* Paperna, 1979 ex *B. docmak* (Sudan). Sclerotized structures. *Abbreviations*: Va, ventral anchor; Vb, ventral bar; Da, dorsal anchor; Db, dorsal bar; HI-HVII, hooks; Mco, male copulatory organ

[Based on 10 flattened and 3 unflattened specimens in GAP; Fig. 4]. Body length 781–838 (815; *n* = 3); greatest width 156–194 (171; *n* = 3). Haptor 100–124 (109; *n* = 3) long, 161–194 (179; *n* = 3) wide. Ventral anchor: a = 27–33 (30; *n* = 10); b = 9–14 (12; *n* = 10); c = 8–11 (9; *n* = 10); x/y = 1.1–1.6 (1.3; *n* = 10). Dorsal anchor: a = 37–43 (40; *n* = 10); b = 8–9 (9; *n* = 10); c = 16–18 (17; *n* = 10); x/y = 0.9–1.3 (1.2; *n* = 10). Ventral bar: d = 47–57 (52; *n* = 10). Dorsal bar: e = 58–73 (64; *n* = 10); f = 26–33 (29; *n* = 10); g = 17–23 (20; *n* = 10); h = 12–15 (13; *n* = 10). Hooks: 7 pairs; *i* = 12–25 (15; *n* = 3): hook I 14–16 (15; *n* = 3); hooks II–V 12–16 (14; *n* = 3); hook VI 21–25 (23; *n* = 10); hook VII 13–15 (15; *n* = 3). Vagina: not observed. MCO: k = 32–35 (34; *n* = 10); l = 29–33 (31; *n* = 10).

### Differential diagnosis

This species was elevated from subspecies status under *Q. clariadis* and adequately redescribed by Kritsky & Kulo [5]. The morphology and measurements of our specimens generally correspond with the redescription and later characterization of this species by Tripathi et al. [7]. Small differences were observed in the morphology of the hooks. However, the shapes of the hooks fall within the variation observed in our series. In individual specimens, the shanks of hooks appear to be more or less robust. The report of *Q. bagrae* by Douëllou & Chishawa [28] is erroneous because their drawings and measurements of the haptoral structures and MCO suggest that these authors were dealing with another *Quadriacanthus* species, most probably with *Q. clariadis*. Their depiction of the sclerotized structures of “*Q. bagrae*” (figure 1) shows a ventral bar with elongated arms (each component is more than twice longer than the total length of the ventral anchor, while it is less than twice longer in *Q. bagrae*) and a dorsal anchor with an elongated bent shaft and short point (in *Q. bagrae*, the dorsal anchor has a short curved shaft and a moderate point), all of which are characters consistent with specimens identified as *Q. clariadis* by El-Naggar & Serag [34], Kritsky & Kulo [5], Tripathi et al. [7], and the present study. Moreover, Douëllou & Chishawa [28] themselves supported our opinion by stating that the hooklets (= hooks) of their specimens were similar to those of *Q. clariadis* rather than to those of *Q. bagrae*. According to our observations, the morphology of *Q. bagrae* and *Q. clariadis* male copulatory organ is very similar; the morphology of haptoral sclerites, however, clearly differs between the two *Quadriacanthus* species.

### Molecular characterization

The sequence of the 18S-ITS1 region of *Q. bagrae* was 921 bp long, of which 515 bp corresponded to the partial 18S rDNA region and 406 bp corresponded to the entire ITS1 region. The sequence of the partial 28S region was 777 bp long. No intraspecific variability was found in 18S-ITS1 or 28S sequences.

***Quadriacanthus clariadis*** **Paperna, 1961**

Syn. *Quadriacanthus clariadis clariadis* Paperna, 1979

***Type-host and locality*** **:** *Clarias gariepinus* (Burchell) (syn. *C. lazera*) (Clariidae), Lake Galilee, Israel [4].

***Host*** **:** *Clarias gariepinus* (present study).

***Localities*** **:** Lake Turkana, Kenya (localities 1, 3); Nile River Basin, Sudan (localities 4, 5) (present study).

***Site in host*** **:** Gill lamellae.

***Other records*** **:** *Clarias gariepinus* (syn. *C. lazera*), Bahr Mouis, River Nile near Zagazig, Egypt [35], Lake Manzala and Demietta Branch, River Nile near Mansoura, Egypt [34], River Nile near Cairo, Egypt [5]; *C. gariepinus*, River Gomti, Lucknow, State of Uttar Pradesh, India [7]; *C. gariepinus*, Nwanedi-Luphephe Dams, Limpopo River System, South Africa [30].

***Voucher material*** **:** MNHN HEL627 (1 specimen; locality 3); MNHN HEL630 (1 specimen; locality 5); MNHN HEL631 (1 specimen; locality 4); MNHN HEL632 (2 specimens; locality 1); IPCAS M-262 (1 specimen from locality 5; 2 specimens from locality 4; 1 specimen from locality 3). Hologenophore: MNHN HEL642 (locality 5).

***Comparative material examined*** **:** Voucher specimen of *Quadriacanthus clariadis* Paperna, 1961 (MRAC 37.160).

***Representative DNA sequences*** **:** 18S-ITS1 rDNA (GenBank acc. no. KX713994) and 28S rDNA (GenBank acc. no. KX685952) (see also Table 2).

### Measurements

**Fig. 5:**
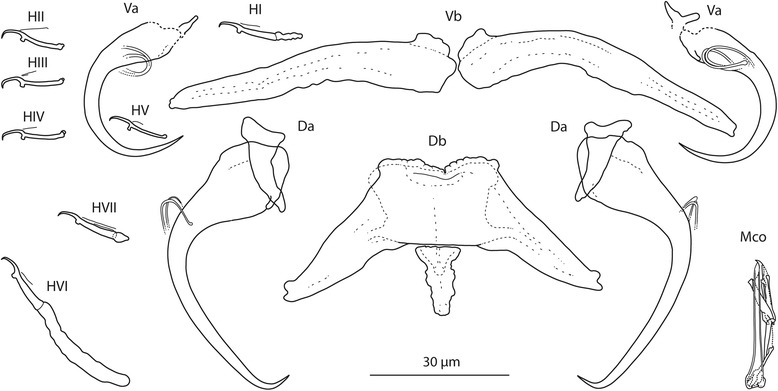
*Quadriacanthus clariadis* Paperna, 1961 ex *C. gariepinus* (Sudan). Sclerotized structures. *Abbreviations*: Va, ventral anchor; Vb, ventral bar; Da, dorsal anchor; Db, dorsal bar; HI-HVII, hooks; Mco, male copulatory organ

[Based on 10 flattened and 3 unflattened specimens in GAP; Fig. 5]. Body length 491–564 (518; *n* = 3); greatest width 89–115 (100; *n* = 3). Haptor 68–119 (89; *n* = 3) long, 113–151 (131; *n* = 3) wide. Ventral anchor: a = 28–31 (29; *n* = 10); b = 10–13 (11; *n* = 10); c = 7–10 (8; *n* = 10); x/y = 1.1–1.9 (1.4; *n* = 10). Dorsal anchor: a = 45–51 (48; *n* = 10); b = 5–7 (6; *n* = 10); c = 15–20 (17; *n* = 10); x/y = 0.9–1.0 (1.0; *n* = 10). Ventral bar: d = 53–64 (60; *n* = 10). Dorsal bar: e = 54–65 (60; *n* = 10); f = 29–37 (32; *n* = 10); g = 13–19 (17; *n* = 10); h = 13–16 (15; *n* = 10). Hooks: 7 pairs; *i* = 12–39 (18; *n* = 10): hook I 18–22 (20; *n* = 10); hooks II–V 12–15 (14; *n* = 10); hook VI 33–39 (35; *n* = 10); hook VII 15–16 (16; *n* = 10). Vagina: not observed. MCO: k = 26–28 (27; *n* = 10); l = 24–27 (26; *n* = 10).

### Differential diagnosis

This species was adequately redescribed by Kritsky & Kulo [5]. Examination of the voucher specimen (MRAC 37.160) showed that our specimens are conspecific with this material. The morphology of the sclerotized structures of our specimens also corresponds to that observed by Tripathi et al. [7]. Molnar & Mossalam’s [35] report of *Quadriacanthus clariadis* contains photographs indicating that the authors found two *Quadriacanthus* species, i.e. *Q. clariadis* and *Q. aegypticus*, but were not able to distinguish between them (see [5]). *Quadriacanthus clariadis* resembles a number of congeners: *Q. aegypticus* El-Naggar & Serag, 1986; *Q. agnebiensis* N’Douba, Lambert & Euzet, 1999; *Q. allobychowskiella* Paperna, 1979; and *Q. longifilisi* N’Douba, Lambert & Euzet, 1999) [27, 33, 36] by its having a ventral anchor with a curved shaft and long point, a dorsal anchor with an elongated bent shaft and short point, and an MCO composed of a straight tapered copulatory tube and an accessory piece with terminal hook(s). The differentiation of *Q. clariadis* and *Q. aegypticus* is provided in the remarks for the latter species. *Quadriacanthus allobychowskiella* is easily separated from *Q. clariadis* by its dorsal anchor having a large accessory sclerite. In *Q. agnebiensis* and *Q. longifilisi*, the hooks of pair VII are markedly longer that those of the corresponding pair in *Q. clariadis*.

### Molecular characterization

The combined 18S-ITS1 sequence of *Q. clariadis* was 920 bp long. This sequence included 514 bp of the partial 18S rDNA region and the complete 406 bp long ITS1 region. The sequence of the partial 28S region was 845 bp long. No intraspecific variability was found in the 18S-ITS1 and 28S sequences.

***Quadriacanthus fornicatus*** **Francová & Řehulková n. sp.**

***Type-host*** **:** *Clarias gariepinus* (Burchell) (Clariidae).

***Type-locality*** **:** Nile River Basin, Sudan (locality 5).

***Other locality*** **:** Nile River Basin, Sudan (locality 4).

***Type-material*** **:** Holotype: MNHN HEL633. Paratypes: MNHN HEL634 (1 specimen); IPCAS M-634 (1 specimen).

***Voucher material*** **:** MNHN HEL639 (2 specimens; locality 4); IPCAS M-634 (2 specimens; locality 4). Hologenophore: MNHN HEL643 (locality 5).

***Site in host*** **:** Gill lamellae.

***Representative DNA sequences*** **:** 18S-ITS1 rDNA (GenBank acc. no. KX713995) and 28S rDNA (GenBank acc. no. KX685953) (see also Table 2).

***ZooBank registration*** **:** To comply with the regulations set out in article 8.5 of the amended 2012 version of the International Code of Zoological Nomenclature (ICZN) [37], details of the new species have been submitted to ZooBank. The Life Science Identifier (LSID) of the article is urn:lsid:zoobank.org:pub:ADCB9E56-E8F1-48B6-AD21-BBA5E52D0B39. The LSID for the new name *Quadriacanthus fornicatus* n. sp. is urn:lsid:zoobank.org:act:6C76112A-B9EA-45E4-9DBB-96B3738A12CF.

***Etymology*** **:** The specific name is derived from Latin (*fornicatus* = arched, vaulted) and refers to the shape of the dorsal anchor shaft.

### Description

**Fig. 6:**
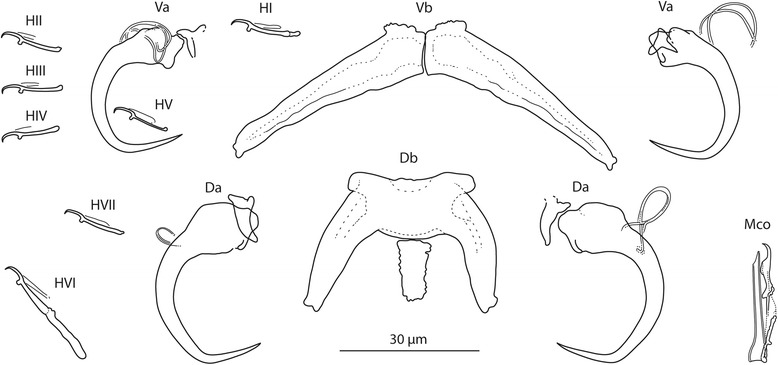
*Quadriacanthus fornicatus* n. sp. ex *C. gariepinus* (Sudan). Sclerotized structures. *Abbreviations*: Va, ventral anchor; Vb, ventral bar; Da, dorsal anchor; Db, dorsal bar; HI-HVII, hooks; Mco, male copulatory organ

[Based on 7 flattened and 2 unflattened specimens in GAP; Fig. 6]. Body length 384–404 (394; *n* = 2); greatest width 75–84 (80; *n* = 2). Haptor 77–84 (80; *n* = 2) long, 84–95 (89; *n* = 2) wide. Ventral anchor with moderate base, curved shaft, long point; accessory sclerite small, with subequal wings; a = 26–31 (28; *n* = 7); b = 11–13 (12; *n* = 7); c = 7–8 (7; *n* = 5); x/y = 1.0–1.3 (1.2; *n* = 7). Dorsal anchor with broad base, curved shaft, long point; accessory sclerite moderate, with poorly differentiated wings; a = 32–39 (35; *n* = 7); b = 12–13 (12; *n* = 7); c = 9–14 (12; *n* = 7); x/y = 0.9–1.2 (1.1; *n* = 7). Ventral bar composed of two elongated components; d = 44–54 (49; *n* = 5). Dorsal bar with small anterior shield; mid-posterior process trapeziform, with uneven margins; e = 42–64 (54; *n* = 6); f = 17–33 (27; *n* = 6); g = 8–14 (12; *n* = 7); h = 9–13 (12; *n* = 7). Hooks: 7 pairs, dissimilar in size; *i* = 12–29 (15; *n* = 5): hook I 13–17 (15; *n* = 7), hooks II–V 12–14 (13; *n* = 5), hook VI 25–29 (27; *n* = 7), hook VII 13–14 (14; *n* = 6). Vagina not observed. MCO comprising copulatory tube and accessory piece; k = 23–29 (26; *n* = 7). Copulatory tube straight; base simple, without thickened margin or flange; l = 22–26 (24; *n* = 7). Accessory piece basally articulated to the copulatory tube in the form of a spike-like structure; medial part lightly sclerotized; distal part a hook-like structure with broader base.

### Differential diagnosis

*Quadriacanthus fornicatus* n. sp. could be confused with *Q. simplex*, a species described on *Heterobranchus isopterus* in Ivory Coast by N’Douba et al. [36], by having nearly identical haptoral sclerites. However, these species are easily differentiated by the comparative morphology of the MCO. The accessory piece of the MCO in *Q. simplex* is noticeably simpler than that in the new species.

### Molecular characterization

The combined 18S-ITS1 sequence of *Q. fornicatus* n. sp. was 912 bp long. This sequence included 493 bp of the partial 18S rDNA region and the complete 419 bp-long ITS1 region. The sequence of the partial 28S region was 847 bp long. No intraspecific variability was found in the 18S-ITS1 and 28S sequences.

***Quadriacanthus mandibulatus*** **Francová & Řehulková n. sp.**

***Type-host*** **:** *Heterobranchus bidorsalis* Geoffroy Saint-Hilaire (Clariidae).

***Type-locality*** **:** Nile River Basin, Sudan (locality 5).

***Other locality*** **:** Lake Turkana, Kenya (locality 3).

***Type-material*** **:** Holotype: MNHN HEL635. Paratypes: MNHN HEL635 (1 specimen); MNHN HEL636 (1 specimen); IPCAS M-635 (1 specimen).

***Voucher material*** **:** MNHN HEL637 (3 specimens; locality 3); IPCAS M-635 (2 specimens; locality 3). Hologenophore: MNHN HEL644 (locality 5).

***Comparative material examined*** **:** Type-specimens of *Quadriacanthus thysi* N’Douba, Lambert & Euzet, 1999 (holotype MNHN 577 HF Tk 94; paratypes 576 HF Tk 93 and MRAC 37416).

***Site in host*** **:** Gill lamellae.

***Representative DNA sequences*** **:** 18S-ITS1 rDNA (GenBank acc. no. KX713996) and 28S rDNA (GenBank acc. no. KX685954) (see also Table 2).

***ZooBank registration*** **:** To comply with the regulations set out in article 8.5 of the amended 2012 version of the International Code of Zoological Nomenclature (ICZN) [37], details of the new species have been submitted to ZooBank. The Life Science Identifier (LSID) of the article is urn:lsid:zoobank.org:pub:ADCB9E56-E8F1-48B6-AD21-BBA5E52D0B39. The LSID for the new name *Quadriacanthus mandibulatus* n. sp. is urn:lsid:zoobank.org:act:AD61A17B-E49C-49B6-B47F-A5CCA2EDD221.

***Etymology*** **:** The specific name is derived from Latin (*mandibula* = an insect mandible; treated as an adjective) and reflects the insect mandible appearance of the dorsal bar process.

### Description

**Fig. 7:**
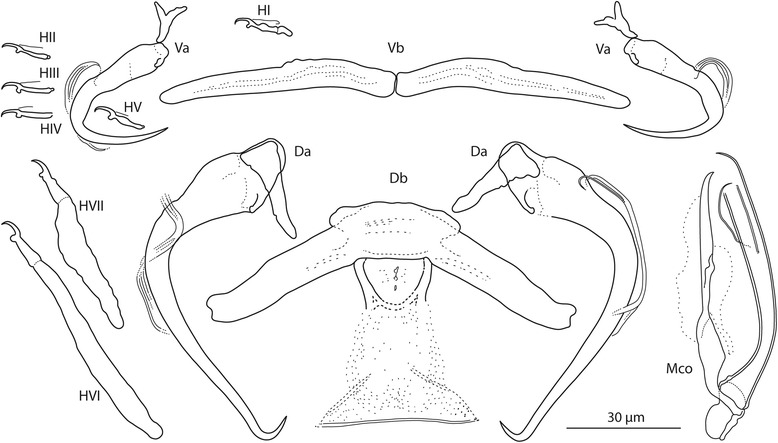
*Quadriacanthus mandibulatus* n. sp. ex *H. bidorsalis* (Sudan). Sclerotized structures. *Abbreviations*: Va, ventral anchor; Vb, ventral bar; Da, dorsal anchor; Db, dorsal bar; HI-HVII, hooks; Mco, male copulatory organ

[Based on 10 flattened and 5 unflattened specimens in GAP; Fig. 7]. Body length 569–840 (734; *n* = 5); greatest width 132–148 (139; *n* = 5). Haptor 140–184 (158; *n* = 5) long, 164–192 (178; *n* = 5) wide. Ventral anchor with narrow base, shaft sharply (at about 90°) bent medially, slightly recurved (poorly differentiated) point; accessory sclerite Y-shaped, with two subequal wing-like processes; a = 28–30 (29; *n* = 10); b = 12–14 (13; *n* = 10); c = 9–15 (13; *n* = 10); x/y = 1.0–1.3 (1.1; *n* = 10). Dorsal anchor with small base, elongated shaft bent (at about 90°) proximally and waved distally, short recurved point; accessory sclerite triangular; a = 72–86 (79; *n* = 10); b = 8–9 (8; *n* = 10); c = 24–27 (26; *n* = 10); x/y = 0.9–1.4 (1.1; *n* = 10). Ventral bar composed of two elongated components articulating medially; d = 54–68 (62; *n* = 10). Dorsal bar broadly V-shaped, with small anterior shield; large mid-posterior process, an insect mandible-like sclerotized structure distally passing into a lightly sclerotized membrane (often with a needle-like distal margin); e = 84–102 (95; *n* = 10); f = 28–41 (34; *n* = 10); g = 12–18 (15; *n* = 10); h = 13–17 (15; *n* = 6). Hooks: 7 pairs, dissimilar in size; *i* = 12–74 (28; *n* = 10): hook I 14–18 (16; *n* = 10); hooks II–V 12–17 (14; *n* = 10); hook VI 67–74 (70; *n* = 10); hook VII 49–56 (52; *n* = 10). Vagina not observed. MCO comprising copulatory tube and accessory piece; k = 70–79 (74; *n* = 10). Copulatory tube a broad slightly curved tube with spoon-like base and subterminal flange; l = 66–71 (69; *n* = 10). Accessory piece articulated to the base of the copulatory tube, with constricted medial part and hook-shaped terminal portion.

### Differential diagnosis

Based on the comparative morphology of the haptoral sclerites, *Q. mandibulatus* n. sp. resembles *Quadriacanthus thysi* described on the gills of *Heterobranchus longifilis* (Agnéby River, Ivory Coast) by N’Douba et al. [36]. The new species differs from the latter species by possessing a lightly sclerotized (poorly differentiated or needle-like) distal part of the supporting membrane of the dorsal bar (the distal part of the supporting membrane is fimbriated in *Q. thysi*) and from all other congeneric species by having a comparatively broad copulatory tube with subterminal flange.

### Molecular characterization

The sequence of the 18S-ITS1 region of *Q. mandibulatus* n. sp. was 879 bp long, of which 500 bp corresponded to the partial 18S rDNA region and 379 bp corresponded to the ITS1 region. The sequence of the partial 28S region was 777 bp long. No intraspecific variability was found in the 18S-ITS1 and 28S sequences.

***Quadriacanthus pravus*** **Francová & Řehulková n. sp.**

***Type-host*** **:** *Clarias gariepinus* (Burchell) (Clariidae).

***Type-locality*** **:** Nile River Basin, Sudan (locality 5).

***Other locality*** **:** Nile River Basin, Sudan (locality 4).

***Type-material*** **:** Holotype: MNHN HEL638. Paratype: IPCAS M-636 (1 specimen).

***Voucher material*** **:** MNHN HEL639 (1 specimen; locality 4). Hologenophore: MNHN HEL645 (locality 5).

***Comparative material examined*** **:** Voucher specimen of *Quadriacanthus numidus* Kritsky & Kulo, 1988 (MNHN 146 HF).

***Site in host*** **:** Gill lamellae.

***Representative DNA sequences*** **:** 18S-ITS1 rDNA (GenBank acc. no. KX713997) and 28S rDNA (GenBank acc. no. KX685955) (see also Table 2).

***ZooBank registration*** **:** To comply with the regulations set out in article 8.5 of the amended 2012 version of the International Code of Zoological Nomenclature (ICZN) [37], details of the new species have been submitted to ZooBank. The Life Science Identifier (LSID) of the article is urn:lsid:zoobank.org:pub:ADCB9E56-E8F1-48B6-AD21-BBA5E52D0B39. The LSID for the new name *Quadriacanthus pravus* n. sp. is urn:lsid:zoobank.org:act:11D623F6-CA7D-434F-AB7C-28D44D17C0A5.

***Etymology*** **:** The specific name is derived from Latin (*pravus* = crooked, distorted, deformed) and refers to the shape of the ventral anchor point.

### Description

**Fig. 8:**
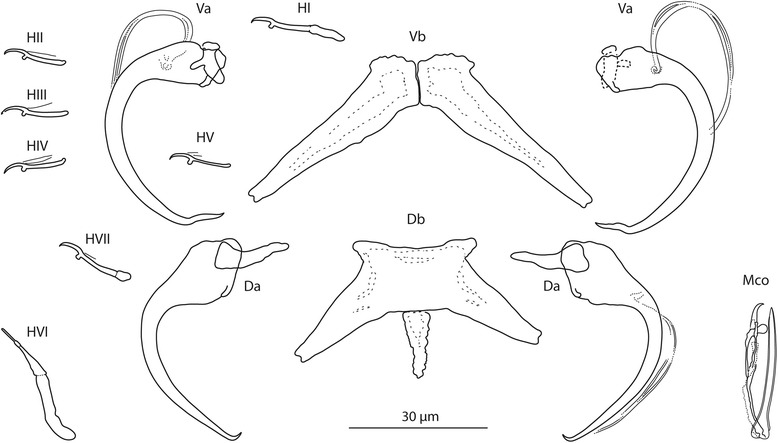
*Quadriacanthus pravus* n. sp. ex *C. gariepinus* (Sudan). Sclerotized structures. *Abbreviations*: Va, ventral anchor; Vb, ventral bar; Da, dorsal anchor; Db, dorsal bar; HI-HVII, hooks; Mco, male copulatory organ

[Based on 4 flattened and 2 unflattened specimens in GAP; Fig. 8]. Body length 580–589 (585; *n* = 2); greatest width 98–144 (121; *n* = 2). Haptor 78–108 (93; *n* = 2) long, 111–162 (136; *n* = 2) wide. Ventral anchor with small base, long evenly curved shaft; doubly recurved (waved) point; accessory sclerite small, wings unequal; a = 39–40 (40; *n* = 4); b = 8–9 (8; *n* = 4); c = 8–10 (9; *n* = 3); x/y = 1.4–1.6 (1.5; *n* = 4). Dorsal anchor with relatively broad base, bent shaft; tiny point; accessory sclerite triangular; a = 41–44 (43; *n* = 4); b = 2–3 (2; *n* = 4); c = 15–17 (16; *n* = 4); x/y = 1.0–1.0 (1.0; *n* = 4). Ventral bar composed of two rapidly tapering components; d = 38–44 (41; *n* = 4). Dorsal bar with small anterior shield; mid-posterior process triangular, with uneven margin: e = 47–53 (50; *n* = 4); f = 25–26 (26; *n* = 4); g = 11–14 (12; *n* = 4); h = 11–15 (13; *n* = 4). Hooks: 7 pairs, dissimilar in size; *i* = 12–29 (17; *n* = 2): hook I 19–21 (20; *n* = 3), hooks II–V 12–14 (13; *n* = 2); hook VI 28–29 (28; *n* = 4), hook VII 16–17 (17; *n* = 4). Vagina not observed. MCO comprising copulatory tube and accessory piece; k = 29–32 (30; *n* = 4). Copulatory tube a short lightly curved tube; length 27–30 (28; *n* = 4). Accessory piece articulated to base of the copulatory tube; proximal part rod-shaped (usually lightly sclerotized); medial part complex; distal part with terminal hook and subterminal pestle.

### Differential diagnosis

*Quadriacanthus pravus* n. sp. resembles the following species by its ventral anchor having a doubly recurved point: *Q. ashuri* Kritsky & Kulo, 1988; *Q. numidus* Kritsky & Kulo, 1988; *Q. papernai* Kritsky & Kulo, 1988; and *Q. gourenei* N’Douba, Lambert & Euzet, 1999 [5, 36]. It differs from *Q. gourenei* and *Q. papernai* by the ventral bar possessing longer (rapidly tapering) components, and is easily differentiated from *Q. ashuri* by having a ventral anchor with a longer shaft. The new species most closely resembles *Q. numidus* in the morphometry of the haptoral sclerites; in particular, the ventral anchor of both species is characteristic by having a relatively small base and markedly long evenly curved shaft, and by lacking a sclerotized vagina. However, *Q. pravus* n. sp. differs from *Q. numidus* in the shape of the accessory sclerite of the dorsal anchor (triangular in *Q. pravus* vs wing-shaped in *Q. numidus*), and in having an MCO characterized by an accessory piece with a terminal hook and subterminal pestle (an accessory piece lamellate in *Q. numidus*). Douëllou & Chishawa [28] reported that the accessory piece of the MCO of their specimens, identified as *Q. numidus,* was slightly different from that described by Kritsky & Kulo [5]. According to Douëllou & Chishawa’s [28] characterization and depiction, it seems that their specimens are conspecific with our specimens (*Q. pravus* n. sp.) rather than with the *Q. numidus* specimens of Kritsky & Kulo [5]. However, because of the poor condition of the slide (MNHN 146 HF), the MCO could not be observed in any of the two voucher specimens. Thus, we hesitate to formally synonymize *Q. numidus* of Douëllou & Chishawa [28] with *Q. pravus* n. sp. at this time.

### Molecular characterization

The sequence of the 18S-ITS1 region of *Q. pravus* n. sp. was 919 bp long, of which 514 bp corresponded to the partial 18S rDNA region and 405 bp corresponded to the entire ITS1 region. The sequence of the partial 28S region was 799 bp long. No intraspecific variability was found in the 18S-ITS1 and 28S sequences.

***Quadriacanthus zuheiri*** **Francová & Řehulková n. sp.**

***Type-host*** **:** *Clarias gariepinus* (Burchell) (Clariidae).

***Type-locality*** **:** Nile River Basin, Sudan (locality 5).

***Other locality*** **:** Nile River Basin, Sudan (locality 4).

***Type-material*** **:** Holotype: MNHN HEL640.

***Voucher material*** **:** MNHN HEL639 (1 specimen; locality 4); IPCAS M-637 (2 specimens; locality 4). Hologenophore: MHHN HEL646 (locality 5).

***Comparative material examined*** **:** Type-specimens of *Quadriacanthus agnebiensis* N’Douba, Lambert & Euzet, 1999 (holotype and two paratypes MNHN 572 HF Tk 89).

***Site in host*** **:** Gill lamellae.

***Representative DNA sequences*** **:** 18S-ITS1 rDNA (GenBank acc. no. KX713998) and 28S rDNA (GenBank acc. no. KX685956) (see also Table 2).

***ZooBank registration*** **:** To comply with the regulations set out in article 8.5 of the amended 2012 version of the International Code of Zoological Nomenclature (ICZN) [37], details of the new species have been submitted to ZooBank. The Life Science Identifier (LSID) of the article is urn:lsid:zoobank.org:pub:ADCB9E56-E8F1-48B6-AD21-BBA5E52D0B39. The LSID for the new name *Quadriacanthus zuheiri* n. sp. is urn:lsid:zoobank.org:act:A725FAF7-9AAF-4156-9647-6AE3EB7A0BCC.

***Etymology*** **:** This species is named in honour to Prof. Zuheir N. Mahmoud of the Department of Zoology, Faculty of Science, University of Khartoum, Khartoum, Sudan, for his valuable and kind assistance during our field campaigns in Sudan.

### Description

**Fig. 9:**
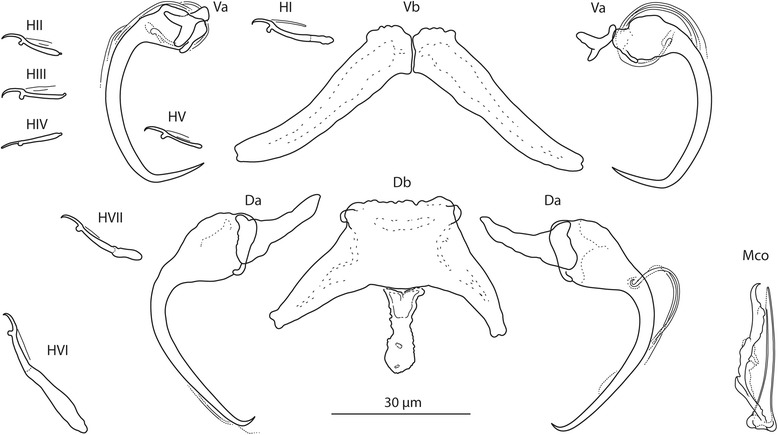
*Quadriacanthus zuheiri* n. sp. ex *C. gariepinus* (Sudan). Sclerotized structures. *Abbreviations*: Va, ventral anchor; Vb, ventral bar; Da, dorsal anchor; Db, dorsal bar; HI-HVII, hooks; Mco, male copulatory organ

[Based on 6 flattened and 2 unflattened specimens in GAP; Fig. 9] Body length 600–690 (645; *n* = 2); greatest width 124–140 (132; *n* = 2). Haptor 95–113 (104; *n* = 2) long, 124–125 (124; *n* = 2) wide. Ventral anchor with moderate base, slightly curved shaft, long point; accessory sclerite small, with subequal wings; a = 37–38 (37; *n* = 6); b = 10–11 (11; *n* = 6); c = 8–11 (9; *n* = 6); x/y = 1.1–1.6 (1.4; *n* = 6). Dorsal anchor with large base, shaft bent proximally, tiny point; accessory sclerite large, triangular; a = 43–47 (45; *n* = 6); b = 2–3 (3; *n* = 6); c = 18–22 (20; *n* = 6); x/y = 1.0–1.3 (1.1; *n* = 6). Ventral bar composed of two elongated components; d = 39–44 (41; *n* = 5). Dorsal bar with small anterior shield; mid-posterior process tongue-like, with uneven margin; e = 42–51 (46; *n* = 5); f = 24–32 (29; *n* = 6); g = 13–18 (15; *n* = 6); h = 11–18 (14; *n* = 5). Hooks: 7 pairs, dissimilar in size; *i* = 12–31 (17; *n* = 5): hook I 17–18 (18; *n* = 6), hooks II–V 12–14 (13; *n* = 5), hook VI 27–31 (29; *n* = 6), hook VII 19–19 (19; *n* = 6). Vagina not observed. MCO comprising copulatory tube and accessory piece; k = 30–34 (32; *n* = 6). Copulatory tube straight to slightly curved; base with thickened margins; l = 28–30 (30; *n* = 6). Accessory piece basally articulated to the copulatory tube; medial part formed as a clamp jaw; hook-shaped termination serving as a guide for distal portion of the copulatory tube.

### Differential diagnosis

Based on the comparative morphology of the haptoral sclerites, *Q. zuheiri* n. sp. most closely resembles *Q. aegypticus* El-Naggar & Serag, 1986, and may also be confused with *Q. agnebiensis* N’Douba, Lambert & Euzet, 1999, a parasite of *Heterobranchus isopterus* from Ivory Coast [27, 36]. *Quadriacanthus zuheiri* n. sp. differs from *Q. aegypticus* by having a noticeably smaller MCO composed of a copulatory tube without basal flange (with flange in *Q. aegypticus*) and simpler accessory piece (i.e. without two medial diverticula and distal hooks). Examination of the holotype and two paratypes of *Q. agnebiensis* showed that *Q. zuheiri* n. sp. differs from the latter species by possessing: (i) a longer ventral anchor with less arched shaft; (ii) a larger accessory sclerite on the part of the dorsal anchor; (iii) shorter and less robust hooks VI and VII; and (iv) an accessory piece with more complex medial part (formed as a lightly sclerotized clamp jaw) and hooked (double hooked in *Q. agnebiensis*) distal termination.

### Molecular characterization

The sequence of the 18S-ITS1 region of *Q. zuheiri* n. sp. was 877 bp long, of which 469 bp corresponded to the 18S rDNA region and 408 bp corresponded to the ITS1 region. The sequence of the partial 28S region was 772 bp long. No intraspecific variability was found in the 18S-ITS1 and 28S sequences.

### Interspecific genetic relationships within genus *Quadriacanthus*

No intraspecific variability was detected for the 18S-ITS1 and 28S regions. The overall K2P mean genetic distance was 10.34% for the 18S-ITS1 sequences and 3.32% for the 28S rDNA sequences. The pairwise genetic distances are presented in Table 3. Among the *Quadriacanthus* species, *Q. clariadis* exhibited the lowest genetic divergence from *Q. bagrae* (1.89% for 18S-ITS1, 0.92% for 28S). *Q. mandibulatus* n. sp. and *Q. fornicatus* n. sp. exhibited the greatest genetic distances (5.87%) for 28S rDNA sequences, and *Q. mandibulatus* n. sp. and *Q. bagrae* represented the most divergent species pair for 18S-ITS1 sequences (13.95%; Table 3).

**Table 3:** Pairwise 18S-ITS1 (below diagonal) and 28S (above diagonal) nucleotide divergences for each observed *Quadriacanthus* spp. using K2P distance (%)

		1	2	3	4	5	6
1	*Quadriacanthus bagrae*		0.92	2.93	4.88	2.12	2.94
2	*Quadriacanthus clariadis*	1.89		2.80	4.74	2.26	2.80
3	*Quadriacanthus fornicatus* n. sp.	6.40	5.85		5.87	2.94	3.76
4	*Quadriacanthus mandibulatus* n. sp.	13.95	13.32	12.57		4.45	4.73
5	*Quadriacanthus pravus* n. sp.	13.48	13.01	12.58	10.05		1.58
6	*Quadriacanthus zuheiri* n. sp.	13.34	12.12	11.67	10.51	4.50	

**Fig. 10:**
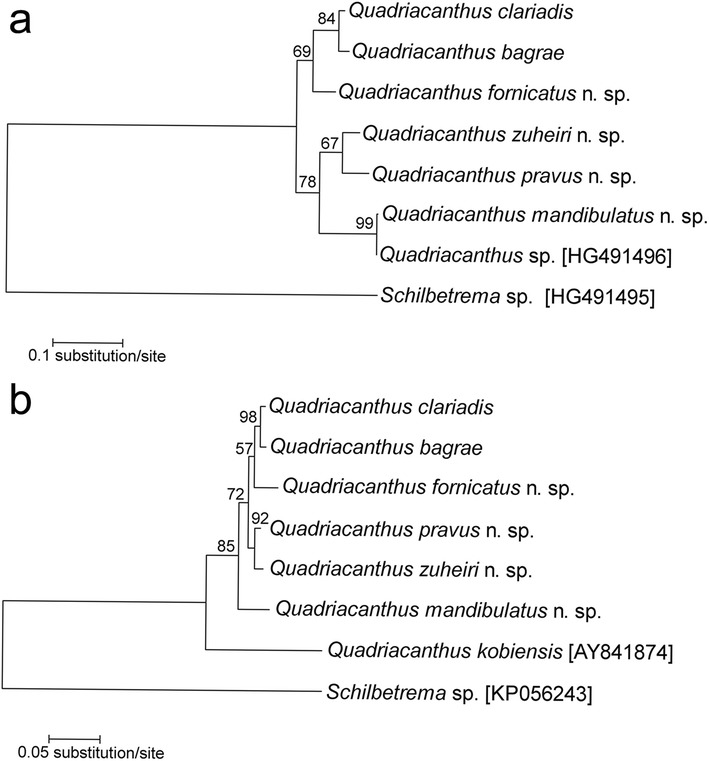
Phylograms for *Quadriacanthus* species parasitizing African freshwater catfishes derived from maximum likelihood analysis using 18S-ITS1 (**a**) and 28S regions (**b**). Bootstrap values ˃ 50% are shown along the branches. Accession numbers for dactylogyrid sequences retrieved from GenBank are shown in *brackets*

An unambiguous alignment of 18S-ITS1 sequences spanned 799 positions, of which 275 positions were variable. The 28S alignment contained a total of 725 bp with 246 variable characters. The phylogenetic trees of *Quadriacanthus* species parasitizing East African freshwater siluriform fishes inferred from 18S-ITS1 and 28S fragments had very similar topologies (Fig. 10). In both gene trees, *Q. bagrae* showed a sister relationship to *Q. clariadis* and *Q. fornicatus* n. sp.; *Q. zuheiri* n. sp. was sister to *Q. pravus* n. sp. For 28S, *Q. mandibulatus* n. sp. formed a separate clade (occupying a basal position); for 18S, *Q. mandibulatus* n. sp. formed one clade with *Q. zuheiri* n. sp. and *Q. pravus* n. sp. Moreover, the phylogenetic analysis of 18S-ITS1 rRNA gene sequences revealed identity between *Q. mandibulatus* n. sp. and *Quadriacanthus* sp. retrieved from GenBank (they differed in one nucleotide). Therefore, we consider this *Quadriacanthus* sp. with the HG491496 sequence, isolated from the airbreathing clariid *Heterobranchus bidorsalis* in Senegal (Šimková, pers. com.), as a representative of *Q. mandibulatus* n. sp.

## Discussion

The geographical distributions and host preferences of species of *Quadriacanthus* suggest an interesting evolutionary history of the group. Species of *Quadriacanthus* have been confirmed as parasites of fishes representing three families, namely the Clariidae, Bagridae (Siluriformes), and Notopteridae (Osteoglossiformes) [2, 3]. Clariid catfishes most likely originated in Asia 40–50 MY ago but contemporary African and Asian species originated from a common ancestor that was present on the Arabian plate about 15 MY ago [38]. From that moment, the ancestral species came back to Asia and colonized Africa probably through brackish water bridges like lagoons [39]. Species of *Quadriacanthus* infesting clariids occur in the freshwaters of Africa, India, Malaysia, Thailand, China and Vietnam [2, 7]. Inasmuch as members of dactylogyrid genera are generally considered highly host-specific (usually confined to members of a single host family), the wide geographical distribution of *Quadriacanthus* spp. on clariid hosts suggests comparatively old host-parasite relationships, i.e. lasting at least 15 MY. On the other hand, formulating a hypothesis on the origin of *Quadriacanthus* species on bagrids (*B. bajad*, *B. docmak* and *B. orientalis*) in Africa is more problematical. Species of *Quadriacanthus* have not been found on bagrids in Asia, although these fishes have occasionally been examined for gill parasites [2]. The family Bagridae was poorly defined until its revision by Mo [40] and de Pinna [41], who established the families Austroglanididae, Claroteidae and Auchenoglanididae for all African genera (except *Bagrus*!) previously considered members of the Bagridae [42]. The well-known Farenholz’ rule states that the natural classification of some parasite groups usually corresponds directly with the natural relationships of their hosts [43]. Indeed, species of claroteids and auchenoglanidids are known to harbour species of *Protoancylodiscoides* Paperna, 1969 and *Bagrobdella* Paperna, 1969, respectively, while those of *Bagrus* are known to be infected with one species of *Quadriacanthus*, i.e. *Q. bagrae* [2].

Some authors (e.g. Brooks & McLennan [44]) believe that monogeneans possess characteristics that perfectly adapt them for surviving numerous host-switching events. Assuming that members of the Clariidae are the ancestral hosts of species of *Quadriacanthus*, the occurrence of *Q. bagrae* (while clearly a member of the genus) on African bagrid hosts probably resulted from host switching. Our phylogenetic reconstruction indicates that *Q. bagrae* is phylogenetically nested within the parasites from *Clarias gariepinus* at a derived position of the tree (Fig. 10). More specifically, *Q. bagrae* from *Bagrus docmak* is a sister species to *Q. clariadis* from *C. gariepinus*. The clade is located at a derived position of the tree, suggesting that *Q. bagrae* (or its ancestor) transferred from clariids to species of *Bagrus* and not conversely. Several studies suggested that such lateral transfer (host switch) can occur both between related host species (e.g. [45]) and even between phylogenetically distant host species [46–48]. Recently, Nack et al. [3] hypothesized that the presence of *Quadriacanthus euzeti* Nack, Pariselle & Bilong Bilong, 2015 on *Papyrocranus afer* (Notopteridae, Osteoglossiformes) is probably the result of a lateral transfer from species belonging to *Clarias* or *Bagrus* which live sympatrically with *P. afer* in Lake Ossa (South Cameroon). Although more data are needed to resolve phylogenetic relationships within *Quadriacanthus*, the occurrence of *Q. bagrae* on *Bagrus docmak* may represent a similar lateral transfer from a species of *Clarias*, probably *C. gariepinus*. *Bagrus docmak* inhabits, among other locations, the Nile River, where it lives in sympatry with *Clarias gariepinus* [9].

Although we cannot verify the accuracy of the identification, *Q. bagrae* was also recorded on *C. gariepinus* by some authors [5, 7]. Because the drawings of the MCO provided by these authors are insufficient for detailed comparison with our specimens, confirmation of the records of *Q. bagrae* on *C. gariepinus* will depend on the collection and evaluation (morphological and molecular) of new parasite material from *C. gariepinus*. It will be interesting to see whether *Q. bagrae* on *C. gariepinus* is a genuine *Q. bagrae* (sensu stricto). If they represent two different species of *Quadriacanthus*, then the occurrence of *Q. bagrae* on *Bagrus docmak* may suggest, at this time, a case of host switching with speciation.

**Fig. 11:**
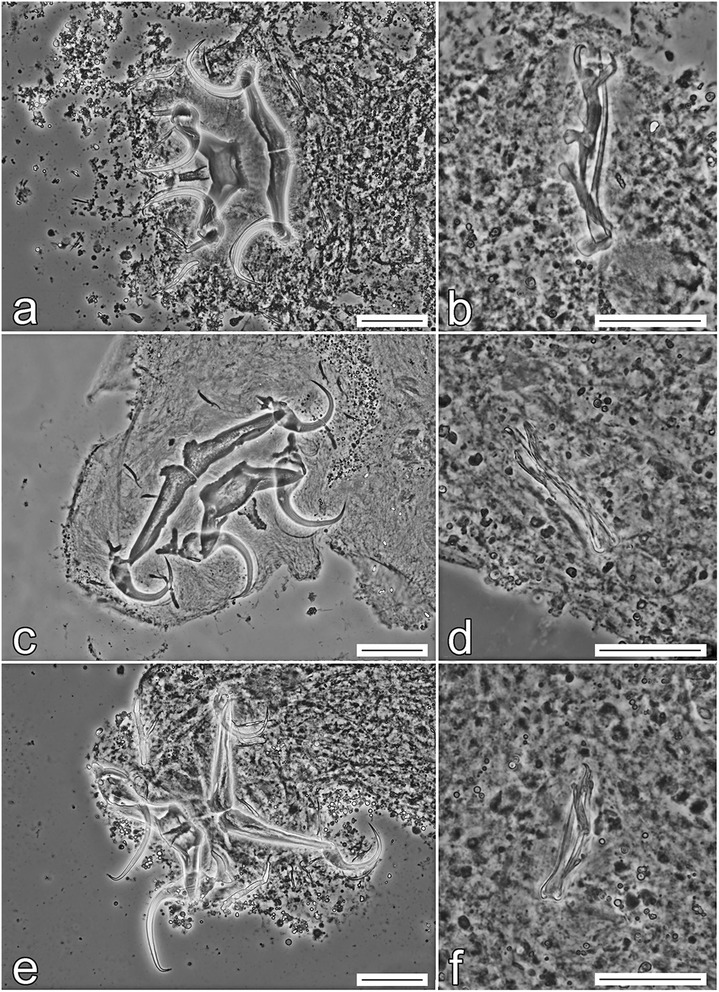
Phase-contrast photomicrographs of the sclerotized haptoral structures and male copulatory organ of *Quadriacanthus aegypticus* (**a**, **b**), *Q. bagrae* (**c**, **d**), *Q. clariadis* (**e**, **f**). *Scale-bars*: **a**, **c**, **e**, 30 μm; **b**, **d**, **f**, 20 μm

**Fig. 12:**
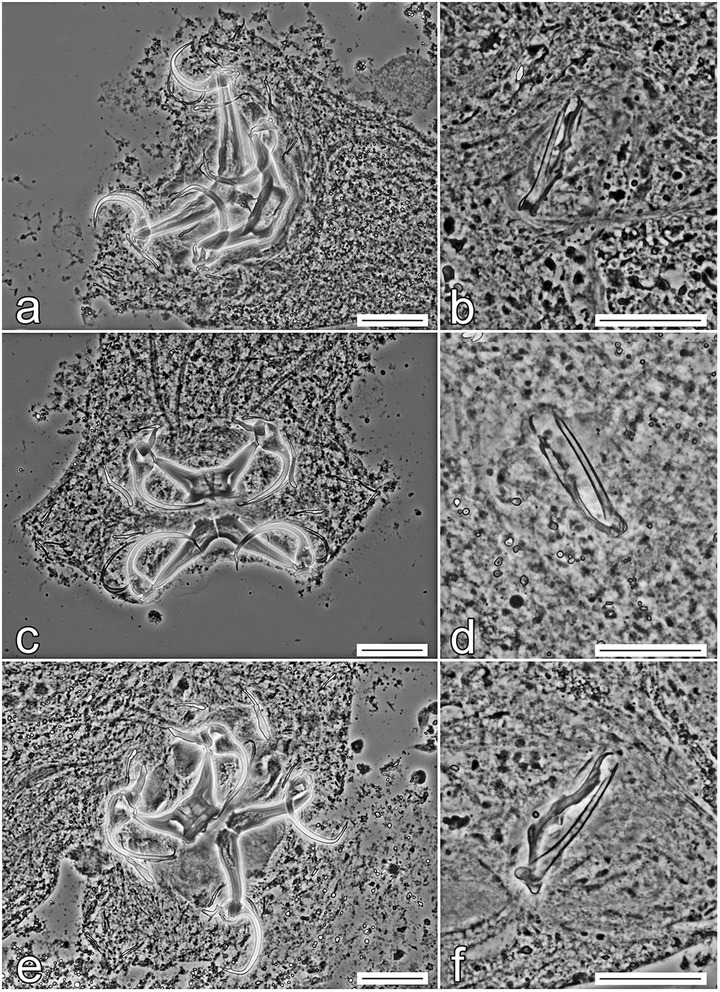
Phase-contrast photomicrographs of the sclerotized haptoral structures and male copulatory organ of *Quadriacanthus fornicatus* n. sp. (**a**, **b**), *Q. pravus* n. sp. (**c**, **d**), *Q. zuheiri* n. sp. (**e**, **f**). *Scale-bars*: **a**, **c**, **e**, 30 μm; **b**, **d**, **f**, 20 μm

**Fig. 13:**
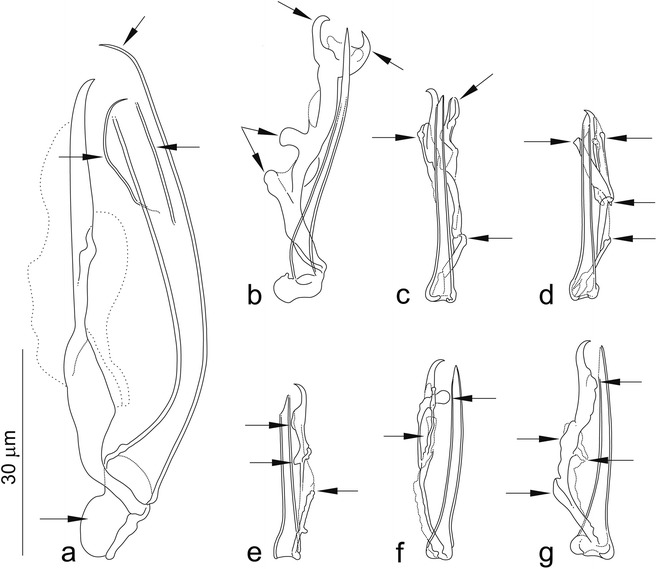
Male copulatory organ of seven representative species of *Quadriacanthus* (characters of interest indicated by arrows). **a** *Q. mandibulatus* n. sp. **b** *Q. aegypticus*. **c** *Q. bagrae.* **d** *Q. clariadis*. **e** *Q. fornicatus* n. sp. **f** *Q. pravus* n. sp. **g** *Q. zuheiri* n. sp*.*

**Fig. 14:**
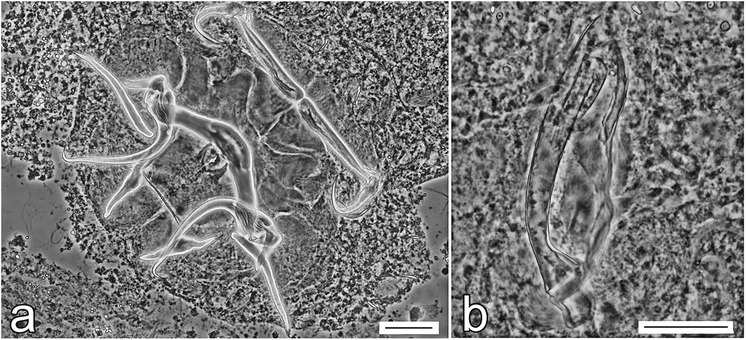
Phase-contrast photomicrographs of the sclerotized structures of *Quadriacanthus mandibulatus* n. sp. **a** Haptoral structures. **b** Male copulatory organ. *Scale-bars*: **a**, 30 μm; **b**, 20 μm

Until now, there were no studies on the genetic characteristics of *Quadriacanthus* spp.; thus, the molecular data presented here represent an important advance in the molecular identification and differentiation of this genus. In our study, molecular characterization is presented for six *Quadriacanthus* species (i.e. for all the species recorded in our study, except *Q. aegypticus*). The interspecific genetic relationships among *Quadriacanthus* spp. observed in this study are congruent with the similarity of the basic morphology of the sclerotized structures, especially of those of the MCO (Figs. 11, 12, 13). The separation of *Q. mandibulatus* n. sp. from the other species corresponds with the different morphology of its copulatory tube. The copulatory tube is terminally enlarged and with a subterminal flange in *Q. mandibulatus* n. sp., while the corresponding structure in all other congeners studied is comparatively small and with an oblique tapering termination (Figs. 13, 14).

## Conclusions

This study suggests that species of *Quadriacanthus* parasitizing catfishes in the Old World provide useful models for the study of biogeography and coevolution. However, future studies are needed that would have to involve the examination of dactylogyrids from a greater number of host individuals and host species from a larger geographical area, the utilization of other monogenean taxa, and the incorporation of a homologous series of host features into the matrix derived from the parasite cladogram.

## Abbreviations

GAP: Glycerine and ammonium picrate
ITS1: Internal transcribed spacer 1
K2: Kimura 2-parameter
MCO: Male copulatory organ
MNHN: Muséum National d’Historie Naturelle
MRAC: Musée Royal de l’Afrique Centrale
PCR: Polymerase chain reaction
